# Livelihood strategies of women entrepreneurs in Indonesia

**DOI:** 10.1016/j.heliyon.2022.e10520

**Published:** 2022-09-02

**Authors:** Achsania Hendratmi, Tri Siwi Agustina, Puji Sucia Sukmaningrum, Mega Ayu Widayanti

**Affiliations:** a Islamic Economics Department, Faculty of Economics and Business, Universitas Airlangga, Surabaya, Indonesia; b Management Department, Faculty of Economics and Business, Universitas Airlangga, Surabaya, Indonesia

**Keywords:** Female entrepreneurship, Sustainable development, Sustainable livelihood, Women entrepreneur, Business strategy, VUCA, COVID-19

## Abstract

Women entrepreneurship is an essential source of economic growth and sustainable development. This study aims to identify the relationship between the five variables of pentagon assets in Sustainable Livelihoods to investigate the survival strategy of women entrepreneurs during COVID-19 in Indonesia. Furthermore, this study aims to build an integrative Women Entrepreneur’s Sustainable Livelihoods model based on volatility, uncertainty, complexity, and ambiguity (VUCA). This study uses Mixed-Methods Investigation by combining the Partial Least Square (PLS) and Group Discussion Forum (FGD). PLS examines the relationship element of women entrepreneurs to build a business strategy and The FGD to support the Livelihoods strategies Model. The purposive sampling technique took the sample of 155 women entrepreneurs (PLS) dan 14 participants (FGD) that matched the sample criteria. The findings demonstrated that women entrepreneurs' livelihood strategies positively linked to their ability to build survival strategies. Second, an integrative model of sustainable livelihood for women entrepreneurs based on the VUCA as an attempt by entrepreneurs to maintain their business during the COVID-19 pandemic. Women entrepreneur sustainability business-based integrative sustainable livelihood model is a solution for women entrepreneurs to survive and develop their businesses. Ability to access five elements of sustainable livelihood Pentagon Assets in Sustainable livelihoods 1) Human Capital; 2) Social Capital; 3) Financial Capital; 4) Physical Capital 5) Intellectual Capital has a positive relationship to the ability to create a business strategy based on VUCA. This integrative Model, compiled based on livelihood strategies and VUCA, can be applied as a survival strategy in women entrepreneurs' businesses dealing with various uncertainties.

## 1 Introduction

Women’s entrepreneurial firms have an essential role in sustainable development goals that are increasingly significant, especially in micro and small businesses. In Indonesia, the small and medium firm sector contributes significantly to employment creation and income. However, the contribution to job creation is very significant; many small and medium businesses encounter challenges in growing and extending their businesses to become large corporations. Women presently account for 49.42% (134 million) of the population in Indonesia, according to the Indonesian Statistics Central Agency (BPS, 2017). According to data from the Ministry of Cooperatives and MSMEs in 2015, Indonesia has over 52 million Small, Medium sectors, with women running 60% of them (Hendratmi and Sukmaningrum, 2018). Women entrepreneurs are primarily found in Micro Small and Medium Enterprises (MSMEs) in Indonesia. For women entrepreneurs, the hurdles of running a small business are considerably more significant. According to Hendratmi and Sukmaningrum (2018), government policies are still missing in growing women entrepreneurs' business motivation and do not focus on women entrepreneurs' traits. Small and medium-sized enterprises (SMEs) are one of Indonesia’s economic levers. MSMEs, according to Law No. 20 (2008), are productive economic businesses carried on by individuals or business entities with a turnover of more than 300 million to 2.5 billion with a total of 5–19 employees, and medium businesses with a turnover of more than 2.5 billion to 50 billion with a total of 20–99 employees (Law of Republic of Indonesia Number 1 of 2008). In Indonesia, MSMEs are quickly expanding in a variety of industries. Men started the majority of the formed families, but most were founded by women (Hani et al., 2012). Women hold 42 percent of Micro and Medium Enterprises (MSMEs) that have been registered as (formal) business entities, according to the International Finance Corporation (IFC, 2016) World Bank Group, and the United States Agency for International Development (USAID). In 2016 52.9 percent of female entrepreneurs in metropolitan areas owned microbusinesses, 50.6 percent owned small businesses, and 34.0 percent owned medium-sized enterprises. Small firms owned by women contributed IDR 443 trillion (USD 36.5 billion) to Indonesia’s GDP, while medium-sized businesses contributed IDR 421 trillion (USD 34.6 billion).

Table 1 shows women’s income in Indonesia; the data shows that female income increases every year. It demonstrates that women play an essential role in a country’s economic development.

**Table 1:** Contribution of Women’s work and income to household income (Indonesia).

Female Income Contribution (Percentage)											
Indonesia	2010	2011	2012	2013	2014	2015	2016	2017	2018	2019	2020
	33.5	34.16	34.7	35.17	35.64	36.03	36.42	36.62	36.70	37.10	37.26

Entrepreneurship is considered a relevant tool for economic growth, and women’s involvement is considerably worldwide (de Vita et al., 2014). Working women are no longer regarded as strange or undesirable, even though work incentive varies (Fahlevi et al., 2019). Women entrepreneurs are vital for developing countries like Indonesia (Nziku et al., 2017). Women entrepreneurs can create jobs, generate commodities, and provide services (Hanson and Blake, 2009; van Praag and Versloot, 2007). Women entrepreneurs have an essential role in family finances and business management (Giones et al., 2020). The ratio of women and men participating in entrepreneurial activity in the state will affect the level of company innovation, according to (Ganna et al., 2021). Women entrepreneurs contributing to the national economy by participating in start-ups and growing small and medium firms (de Vita et al., 2014; Grint, 2020; Gümüsay, 2015) can mimic the successes of gender equality.

The factor that motivates women to pursue entrepreneurship differs from that which motivates males (Kirkwood, 2009). Men are encouraged to start their businesses, whereas women want the flexibility to balance work and family life or help others (Danish and Smith, 2012). Women entrepreneurs are community groups to improve family welfare while also contributing to the national economy. According to a recent study, women entrepreneurs in poor or less-developed nations have stronger self-confidence than women in developed countries. Becoming an entrepreneur is the only way to get employed and keep the home operating smoothly (de Vita et al., 2014). Hisrich et al. (1997) looked into why women launched enterprises and motivated them. The primary motivations for beginning a new business are “push” causes such as frustration and boredom in their former professions and “pull” considerations such as autonomy. Young women with employment possibilities, growing women with external restraints, and consolidated women with a trunked career are all described by Avolio (2012).

Women entrepreneurs have significant hurdles in maintaining their businesses. Business sustainability is a process and set of actions that ensure a company’s long-term viability (Scott, 2017). Many challenges, hurdles, and possibilities for sustainability can be adjusted by mature business procedures, which are a fundamental aspect of entrepreneur ability (Schaltegger and Wagner, 2011). Regardless of the issues and obstacles, women, entrepreneurs must develop ways to manage their businesses to raise income and meet the demands of their families (Muhamad et al., 2017). Because women entrepreneurs have distinct qualities, obstacles, and requirements, this research sustainable livelihood method is employed as a proxy for company sustainability for women entrepreneurs. Environmental, economic, political, and cultural factors connect livelihoods to larger national, regional, and global arenas, making them more than just a local occurrence (Laszlo and Laszlo, 2002).

This study employs five significant human capital, social capital, intellectual capital, financial capital, and physical capital, as the primary foundation in the livelihood plan, based on the prior discussion of women entrepreneurs' traits and motivation. COVID-19 has recently altered the commercial landscape. According to the Secretary of the Ministry of Cooperative and Small Medium Enterprises, 90 percent of small and medium enterprises have ceased operations, and 50 percent are experiencing financial difficulties. This epidemic also impacts other elements; 29,12 billion people have lost or reduced working hours (BPS, 2020). The decline of MSMEs, the most significant contributor to Indonesia’s economic growth, creates a new financial dilemma. The poverty rate increased from 9.78 percent in September 2019 to 9,78 percent in March 2020. The outbreak has the hardest hit the tourist and ancillary industries.

Meanwhile, most female entrepreneurs run a business in the supportive sector (BPS, 2020). The COVID-19 virus, which has spread worldwide, has imposed restrictions on various economic operations, resulting in financial losses (Zhang et al., 2020). Many small firms have been impacted by entrepreneurial ecosystems, notably during the COVID-19 epidemic, as living in the new economy is difficult (Rashid and Ratten, 2021). The importance of entrepreneurs in supporting local industries in any economy is generally recognized, and they are often seen as an economy’s lifeline during difficult times (Budhwar and Cumming, 2020). These COVID-19 conditions and their impact on women entrepreneurs' sustainable livelihoods necessitate academic research that can produce strategies for companies and governments to cope with change (Ratten, 2020), particularly for women entrepreneurs and business strategy ensure their business’s long-term viability.

Enterprises must develop the ability to adapt to environmental changes in response to the constantly changing internal and external environment to survive (Kumar and Rao, 2015; Ma et al., 2020; Odlin, 2019; Pigola et al., 2022). The VUCA is a relevant proxy for highlighting a volatile, uncertain, complex, and ambiguous world in a business’s survival strategy. The real issue is re-imagining the company with that knowledge (Fenton et al., 2019). The corporate environment is fast evolving nowadays, and it appears to be prone to volatility, uncertainty, complexity, and ambiguity, dubbed the “VUCA” period (Lawrence, 2013). The acronym VUCA has been used worldwide to describe today’s operational environment, which is both demanding and developing. Women’s entrepreneurial skills and abilities are no longer sufficient to help their enterprises succeed. They also require greater agility and developing strategic, complicated critical-thinking skills (https://hbr.org/2014/01/).

During COVID-19, however, the most productive businesses of women entrepreneurs were put in jeopardy, and many failed to develop into viable business units. Not just in the industry (Turner and Akinremi, 2020b) but also in socioeconomics (Fernandes, 2020; Nicola et al., 2020a, 2020b), finance (Goodell, 2020), and supply chain (Nicola et al., 2020a, 2020b). The impact of the pandemic on SMEs is divided into three main factors: first, large firms have more significant financial resources than SMEs, which are often described as having limited resources; second, because SMEs operate across a wide range of sectors, failure among SMEs has the potential to impact daily life, as SMEs play an essential role in terms of the local employee; and third, SMEs have unequal job creation, and SMEs will be critical to rebuilding post-crisis (Turner and Akinremi, 2020a).

Based on this phenomenon, We employ a mixed-method approach in this study to explain how women as entrepreneurs use human capital, financial capital, physical capital, natural capital, and social capital to survive in volatile, uncertain, complicated, and ambiguous environments. Because PLS is used to explain the relationship between latent variables, we employed it as a hypothesis test to find out if there is a link between livelihood strategies and survival strategies for women entrepreneurs using VUCA as a proxy (Chin and Newsted, 1999). This study used Focus Group Discussion (FGD) to construct a model based on livelihood and survival strategies and strengthen the Partial Least Squares (PLS) theory.

Research is needed to develop a women’s entrepreneurial process model to encourage productive businesses' independence and long-term viability during COVID-19. Images of women entrepreneurs in Indonesia using the sustainable livelihoods method as a proxy for business sustainability, notably in women-owned small and medium businesses (SMEs). Statistics Indonesia defines small and medium enterprises (SMEs) as businesses with five to 19 employees. On the other hand, a medium-sized company has 20 to 99 people (BPS, 2013). It increased the urgency of research to develop an integrative model for women entrepreneurs with five pentagrams of sustainable livelihoods in the event of a pandemic.

## 2 Related literature

### 2.1 Women entrepreneur

Women entrepreneurs engage in all aspects of entrepreneurship, take risks, and discover opportunities in their surroundings to combine resources in novel ways to benefit their businesses. Home-based businesses that produce Micro, small and medium enterprises (MSMEs) or formal and informal sectors are common among women entrepreneurs. Lewis (2020) went on to say that, like males, women have the freedom to choose, develop, and communicate their entrepreneurial identities. Women entrepreneurs must perform the same tasks as male entrepreneurs, such as seeking out new business opportunities, managing risk, introducing innovations, coordinating, caring for administration, and controlling the business to effectively and adequately lead all aspects of the company (Gümüsay, 2015). Many open innovation policies included women-owned businesses (Meng et al., 2021). Furthermore, women’s entrepreneurship aids in exploring the emancipatory potential of the company, promoting gender equality, and discovering one’s own identity through professional accomplishments (de Vita et al., 2014).

### 2.2 Livelihood strategies

The term “sustainable livelihood” was coined in the early 1990s to describe a notion developed to comprehend better hunger and food shortages in the 1980s (Bhuiyan et al., 2012). Department of International Development (DFID) also launched an action project in 1997 that supports one sustainable livelihood, and one of the aims is meant to accomplish poverty elimination (Bhuiyan et al., 2012). This strategy, according to DFID, would be an excellent way to help people attain long-term livelihood since it is people-centered and emphasizes that we all have a responsibility to each other (DFID, 1999). Sustainable livelihood approaches are primarily asset identification, according to Carney (2004). All components interact, including vulnerability level, policy, and institution. Physical, social, economic, and environmental elements or processes that raise an individual’s susceptibility, a community’s asset, or a system’s vulnerability to the impact of disasters are characterized as vulnerability (Uni/ISDR 2004). The adequate assets form a stable social-political structure and process, allowing people to earn more money, improve their well-being, reduce vulnerability, and improve sustainability (Fauziyanti and Hizbaron, 2020). In a word, livelihood strategy refers to a person’s choice and variety of activities to meet their livelihood needs. A livelihood strategy is a collection of actions and alternative options utilized by an individual or a group to achieve prosperity due to the technique used to live their livelihoods (Illu et al., 2021).

### 2.3 Pentagon assets

The Department of International Development has recognized the following Pentagon assets in sustainable livelihoods: (a) Human Capital; (b) Intellectual Capital; (c) Social Capital; (d) Financial Capital; (e) Physical Capital (DFID, 1999). Human capital refers to a person’s skills, knowledge, and abilities that influence their ability to think and act creatively. Individual-related resources (in the human nodes) are referred to as human capital (Kungwansupaphan and Leihaothabam, 2016). Human capital (knowledge and labor or the power to command labor) is required to utilize any of the four other types of assets, in addition to having intrinsic worth (DFID, 1999). Financial capital refers to the capital basis (cash, credit or debit cards, savings, and other economic assets) required to carry out any livelihood strategy (Krantz, 2001). Financial capital refers to the financial resources that people have access to (such as savings, credit, regular remittances, and pensions), which provide them with a variety of living options (Ahmed and Wahid, 2011). Physical capital is made up of the capital developed throughout the economic production process and the basic infrastructure and producer products required to sustain life (Ahmed and Wahid, 2011; Kabir et al., 2012). According to (Kabir et al., 2012), social capital is the ability to make decisions, interact with the environment, be satisfied with their own business, and deliver the best results. The term “intellectual capital” refers to the accumulation of intangible assets. From a variety of perspectives, social capital is the same as resources. Individuals and business networks bring ideas, business possibilities, financial capital, power, emotional support, goodwill, trust, and cooperation (Baker, 2000). Intellectual capital is an intangible resource that contributes to the organization’s strategic value (Diez et al., 2010). Intellectual capital is becoming a hot topic among businesses that profit from innovation and knowledge-intensive services. Knowledge assets that can be transformed into value are referred to as intellectual capital (Edvinsson and Sullivan, 1996). Cabrita and Bontis (2008) define intellectual capital as “the ability to connect various sets of expertise, experience, and skills within and beyond the company?”

### 2.4 Pandemic COVID-19 as major vulnerability

During the COVID-19 epidemic, human beings' inability to pursue fairways of asset acquisition made it clear that susceptible firms were placed in an unequal condition of well-being. With the emergence of COVID-19, vulnerability has increased, underscoring the need to assure equal access to fundamental assets (Kesar et al., 2021). The state of the nation’s small companies appears to be grim in the aftermath of the coronavirus outbreak. In this ever-changing and uncertain environment, businesses are seeking to be constructive. Small enterprises, labor, and business revenue are most affected by the Coronavirus and the least financially resilient.

### 2.5 VUCA as a proxy of survival strategy

Volatility, uncertainty, complexity, and ambiguity (VUCA) are acronyms for volatility, uncertainty, and ambiguity. Conflict is inherent and unexpected because threats are dispersed and ambiguous, and material and manpower resource limits might hamper defense capability. A volatile situation is one in which there is an unstable or unexpected state, a significant lack of knowledge, or uncertainty regarding the outcome of events (N. Bennett and Lemoine, 2014). It’s also a statistical measure that describes the magnitude of changes (Mack et al., 2015). When a variable of interest cannot be measured directly, a proxy is utilized (Lewis-Beck et al., 2003).

i. Volatility is a characteristic that frequently changes quickly and dramatically (Kaivo-oja and Lauraeus, 2018). The turbulence that occurs more often than in the past is known as volatility (Kaivo-oja and Lauraeus, 2018).
ii. Uncertainty can be defined as a situation in which there is a lack of knowledge about whether a particular event is significant enough to be a meaningful cause (N. Bennett and Lemoine, 2014). Because uncertainty stems from a lack of knowledge, it can be alleviated by allocating more resources to activities that cross boundaries (N. Bennett and Lemoine, 2014).
iii. The term “complexity” or “difficult conditions” refers to a specific response unrelated to the other VUCA components.
iv. Ambiguity is defined as a situation in which the nature of cause-and-effect relationships is uncertain (N. Bennett and Lemoine, 2014). When it comes to making business decisions, there is a lot of ambiguity. There are frequently multiple options and no analytical process to choose the best one (Mack et al., 2015). Ambiguity is characterized by a lack of clarity and difficulty comprehending the situation precisely (Kaivo-oja and Lauraeus, 2018).

In this research, we use VUCA as a proxy for survival strategies. Proxy is a variable used when the variable of interest cannot be measured directly (Lewis-Beck et al., 2003). VUCA is a condition that can be conceptualized as a complex system where volatility and uncertainty are observable and could lead to situations of ambiguity (Mack and Khare, 2016).

### 2.6 Livelihood strategies for women entrepreneurs based on VUCA as a profor of survival strategies

The three essential components of a sustainability plan (Baumgartner and Ebner, 2010) include an economic dimension, which consists of the activities required for a corporation to continue operating in the market. Innovation, teamwork, and information management are all vital company functions for creating economically valuable products or services. The most successful sustainability strategies are fully incorporated into a company’s entire design and require changes to the organization’s fundamental rationale (Baumgartner and Ebner, 2010).

**Figure 1:**
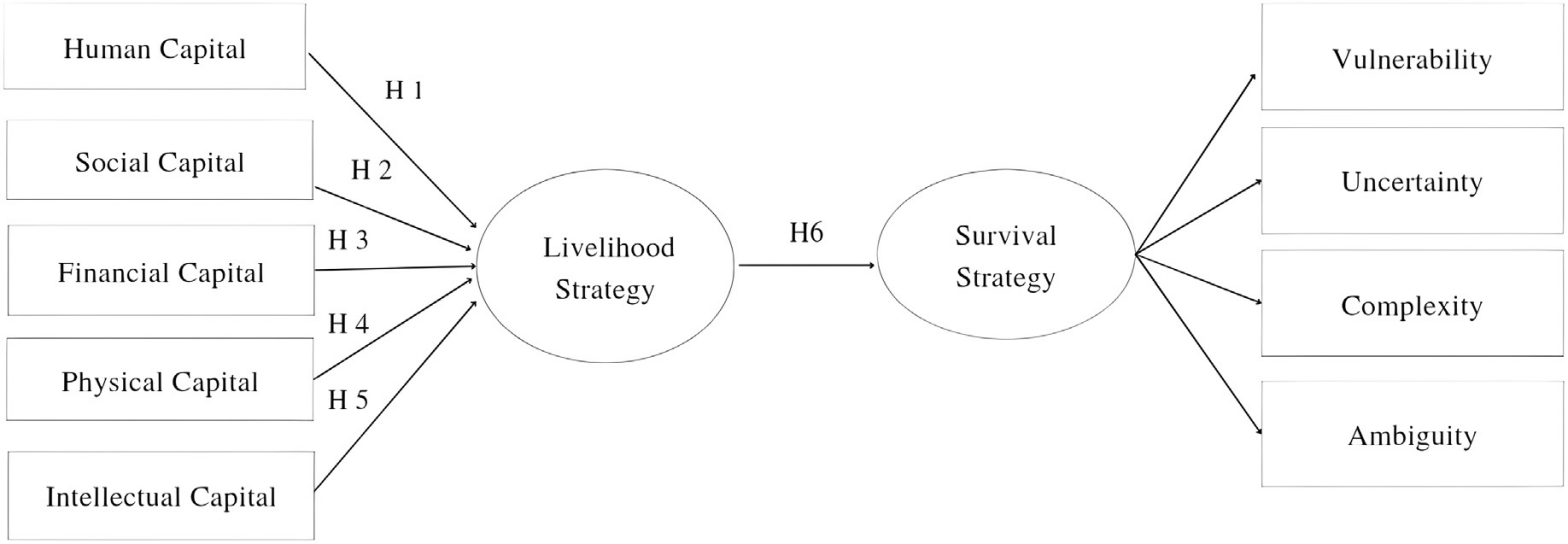
Conceptual framework.

The hypothesis in this study was formed from the Conceptual Framework in Figure 1. The relationship between variables in each variable can be seen below:

#### 2.6.1 Human capital and livelihood strategies

According to the literature, several capital determinants have a crucial impact on women’s entrepreneurship. Human capital is an essential factor influencing the entrepreneurial process. According to (Kungwansupaphan and Leihaothabam, 2016), it can improve the ability to perform entrepreneurial activities, capitalize on future opportunities, gain other resources such as financial and physical capital, and aid in the accumulation of knowledge and skills. A previous study has shown that human capital is positively associated with small business success (Colombo and Grilli, 2010). Entrepreneurs with more human capital have a competitive advantage when supporting their firms in addressing problems, adjusting to new technologies, and applying new technology (Sallah and Caesar, 2020).

H1 Human Capital significantly affects livelihood strategies

#### 2.6.2 Social capital and building livelihood strategies

Individuals' actions are facilitated by social capital in terms of knowledge exchange, capital sharing, and risk reduction, and it leads to business contacts and advisors, which are regarded as crucial in conducting entrepreneurial activity. As a result, social capital is an essential source of support for women who want to start their businesses (Kungwansupaphan and Leihaothabam, 2016). Because they rely on the network to communicate challenges and success stories of women-owned enterprises, social capital has aided them in gaining support during times of business crisis (Sallah and Caesar, 2020).

H2 Social capital significantly affects livelihood strategies

#### 2.6.3 Financial capital and livelihood strategies

Financial capital is critical for a firm, especially now that the economy is slowing and people are more conservative with their spending. Owners must find a way to escape insolvency due to the rising expense of living. Businesses rely on financial resources to invest, expand, and grow, according to Ngek (2016); nevertheless, SMEs have poor levels of financial literacy and financial capital availability on average. Financial capital, such as savings, credit, and debt (formal and informal), remittances, pensions, and wages, is considered one of the most critical assets to support any livelihood activity (Baffoe and Matsuda, 2018).

H3 Financial capital significantly affects livelihood strategies

#### 2.6.4 Physical capital and livelihood strategies

Physical capital is used to achieve organizational objectives. Input, behavioral, and output control are three control system aspects, according to the theory. This notion should also be used to manage physical capital. Physical input would be carefully considered, people’s behavior using physical assets or resources would be carefully controlled, and physical utility output would be carefully evaluated by organizations with a high degree of physical resource control. The good influence of Physical Capital on building a business strategy-based VUCA can be shown by using this method (Usman and Wirawan, 2021).

H4 Physical capital significantly affects livelihood strategies

#### 2.6.5 Intellectual capital and livelihood strategies

Previous research has also underlined the importance of the relationship between intellectual capital and a firm’s competitive advantage and survival (Buenechea-Elberdin, 2017). have looked into the relationship between intellectual capital, innovative activities, and competitiveness in businesses. According to Johnson et al. (2013), intellectual capital has a favorable impact on a company’s performance, survival, and growth.

H5 Intellectual capital significantly affects livelihood strategies

#### 2.6.6 Livelihood strategies and survival strategy

The term strategy signifies an integrated set of organizational actions to attain lasting success in a corporate context (Krishnan et al., 2021). It promotes an organization’s growth and ensures its long-term viability (Gupta et al., 2021). Survival strategies aim to avoid failure or death (Krishnan et al., 2021; Shepherd et al., 2000). During the crisis, businesses should develop survival plans to fulfill their strategic goals and future growth (Bourletidis and Triantafyllopoulos, 2014; Dahles and Susilowati, 2015; Prohorovs, 2020). Many factors have been discovered to influence business survival, including the technological intensity of the sector (Aghion et al., 2001), profitability, and financial constraints (Bellone et al., 2008; Headd, 2003). The latent variable business and patent guidance, which comprises consultants, lawyers, patent councils, and management teams, positively affected firm survival. A firm’s capacity to attract funding, achieve development and profitability, and assure survival is also influenced by the attitudes and motivation of its founders and managers. The relevance of business networks and entrepreneurial business behavior and rivalry in the survival of new technology-based enterprises (Woldehanna et al., 2018).

H6 Livelihood strategies significantly affect Survival strategy

## 3 Methodology

### 3.1 Research design

Design of the study PLS and FGD were used in this mixed-methods investigation. Mixed-methods research enables researchers to compensate for the weaknesses of one methodology with the strengths of the other to reach the best outcome and demonstrate validity (Creswell and Plano, 2011). We utilize PLS to look for partial predictions between livelihood and survival strategies in this work. We use PLS to eliminate assumption assumptions from regression, ensuring that the data is distributed and multicollinearity is not an issue (Ghozali and Latan, 2015). PLS can test poor data, such as small samples or other difficulties. PLS can also explain and corroborate a theory by explaining the relationship between variables (prediction) (Chin and Newsted, 1999). The closed questionnaire was distributed to MSME entrepreneur communities in 34 provinces in Indonesia. A total of 155 respondents participated because they met the author’s criteria of having a monthly turnover of 50 million rupiahs and having been in business for at least three years. All of the respondents in this research approved of this study. The participant gave informed consent to fill out the questionnaire, and they were advised that the information was being collected solely for educational purposes. PLS can answer the purpose of how women entrepreneurs respond to VUCA with the capital they have and used it for hypothesis testing. As is well known, pandemic COVID-19 poses a significant challenge to the economy’s wheels, large or small. Entrepreneurs must rethink effective and efficient techniques to stay afloat in the market. Entrepreneurs face the danger of asset liquidation, capital shortages, and insolvency in the event of a pandemic.

We utilize FGD to develop the Model, Focus Group Discussion (FGD) is used in this study to elicit in-depth information from experts about their expertise, attitudes, and perceptions and develop new model tactics that would help them thrive in the market (Hennink, 2014). Focus Group Discussions (FGD) were held to construct a livelihood and survival strategies model using VUCA as a proxy. The 14 participants in the Focus Group Discussion represent a variety of MSME Women’s entrepreneur founders and organizations in Indonesia in various industries and have been in operation for three years. Purposive sampling was used to choose the focus group discussion members to fulfill the authors' expectations. Thus the answers could vary widely and enrichen the understanding of this research.

### 3.2 Measures

Five indicators from livelihood strategies were included in the analytical framework and approach. Financial, physical, social, and human capital are included (Carney, 2004; Soussan et al., 2000), with intellectual capital replacing natural capital. Endogen constructs are used when latent variables are solely used as dependent variables. Survival strategies are endogenous constructs in this investigation (Sarstedt et al., 2017). When latent variables are used solely as independent variables, they are called Eksogen constructs. Exogenous constructions in this study are Livelihood strategies (Hair et al., 2017). Tables 2 and 3 provide indicators of livelihood and survival strategies, respectively, using VUCA as a proxy.

**Table 2:** Indicators of Women entrepreneur Livelihood Strategies.


Human Capital (HC)	Individuals' talents, expertise, and abilities influence them to think and behave in novel ways. Human capital is the individual-related resources (in the human nodes) (Kungwansupaphan and Leihaothabam, 2016)
Social Capital (SC)	an increase in social prestige, the ability to make decisions, cooperate with the environment, satisfaction with their own business, and provide the best results. Intellectual capital refers to the collection of intangible resources (Kabir et al., 2012)
Physical Capital (PC)	the economic production process and the basic infrastructure and producer goods needed to support livelihood (Ahmed and Wahid, 2011; Kabir et al., 2012)
Financial Capital (FC)	the financial resources available to people (whether savings, supplies of credit, regular remittances or pensions) and provides them with different livelihood options (Ahmed and Wahid, 2011)
Intellectual Capital (IC)	creating and supporting connectivity between all expertise, experience, and competencies inside and outside the organization (Cabrita and Bontis, 2008)

**Table 3:** Indicators of Survival Strategy based on VUCA as a proxy.


Volatility (V)	a quality that changes frequently is fast and significant (Kaivo-oja and Lauraeus, 2018)
Uncertainty (U)	a situation characterized by a lack of knowledge because of whether a particular event is significant enough to constitute a meaningful cause (N. Bennett and Lemoine, 2014)
Complexity (C)	a distinct response utterly separate from those necessitated by the other components of VUCA. (N. Bennett and Lemoine, 2014)
Ambiguity (A)	a situation where there is doubt about the nature of cause-and-effect relationships. (N. Bennett and Lemoine, 2014)

### 3.3 Data collection

The types of data used in this study are primary and secondary data. Preliminary data were obtained from the questionnaire. The questionnaire was distributed to SME women entrepreneurs from 34 provinces in Indonesia. A sample of 450 questionnaires was sent to target SMEs, following the stratified purposive sampling technique, of which a complete 155 responses were duly received. We use purposive sampling, which is suited to this research because the sample unit is selected primarily on personal judgment or convenience. The probability of any particular member of the population being chosen is unknown (Zikmund, 2013). The respondents' profile (Table 4) shows that the participants were 33.55 percent between 30 and 35 years of age and 23.23 percent between 26 and 29 years of age. Due to FGD’s purpose, 14 FGD participants have agreed to have their names published. Meanwhile, secondary data uses data sources such as the Indonesian Central Statistics Agency (BPS), the Ministry of Cooperatives, and MSMEs (Small and Medium Enterprises) and relevant reports.

**Table 4:** Respondents characteristic.

Domicile		
Province	Respondents	Percentage
East Java	38	24,52%
Central Jakarta	28	18,06%
West Java	27	17,42%
Central java	17	10,97%
Banten	8	5,16%
North Sumatera	5	3,23%
South Kalimantan	5	3,23%
Jambi	4	2,58%
Others	23	14,84%
Age	Respondents	Percentage
20–25	32	20,65%
26–29	36	23,23%
30–35	52	33,55%
36–39	18	11,61%
40–45	9	5,81%
>45	8	5,16%
Spending	Respondents	Percentage
<USD 100	20	12,90%
USD 101–200	70	45,16%
USD 201–300	24	15,48%
USD 301–500	26	16,77%
USD 501–750	8	5,16%
>USD 750	7	4,52%
Education	Respondents	Percentage
SD/Elementary	8	5,16%
SMP/Junior High	10	6,45%
SMA/K/High School	73	47,10%
D1/Diploma 1 Year	1	0,65%
D2/Diploma 2 Year	1	0,65%
D3/Diploma 3 Year	12	7,74%
S1/Bachelor	46	29,68%
S2/Master	2	1,29%
S3/Doctoral	1	0,65%
Other	1	0,65%

### 3.4 Statistical method

This research applies the system of Partial Least Square using smartPLS professional 3.0 as a computer statistical tool to process the data collected with the questionnaire and adhere to the planned objectives. Increasing attention to the validity of constructs in general and a more rigorous evaluation of the measurement properties of constructs have prompted procedures based on models of structural equations of latent variables to be considered in the literature as suppliers of tests of reliability, convergent validity, and discriminant validity on the construct.

## 4 Data result

### 4.1 Result

The questionnaire is spread to 450 respondents. Only 155 respondents are qualified for this research criterion. Women entrepreneurs run their business for a minimum of 3 years, and their business has a turnover of 50 million rupiah/month. The sample was collected using a non-probability sampling method, and the respondents came from almost every province of Indonesia. The domicile of the respondents comes from all of Indonesia province. Most respondents come from east java, DKI Jakarta, west java, and central java. Table 4 shows individual needs expense categories into six groups. It shows the needs of the entrepreneur’s life as a human being. The table appears the sample of respondents who mostly spent 101–200 USD where the models conducted in the study have been following the category of SMEs. Based on this questionnaire, it is known that 47,10 percent of respondents graduated from High School, and 29,68% graduated with bachelor’s degrees. This data indicates that female entrepreneurs in Indonesia already have an excellent educational background to build their knowledge.

Results from Partial Least Square (PLS) The PLS program used is SmartPLS Professional 3.0. The PLS consists of two stages; The first stage is testing the outer Model. Next is to test the inner Model. This stage aims to test the hypothesis to ensure an influence between variables. Testing is done using the t-test.

#### 4.1.1 Outer model testing phase

**Figure 2:**
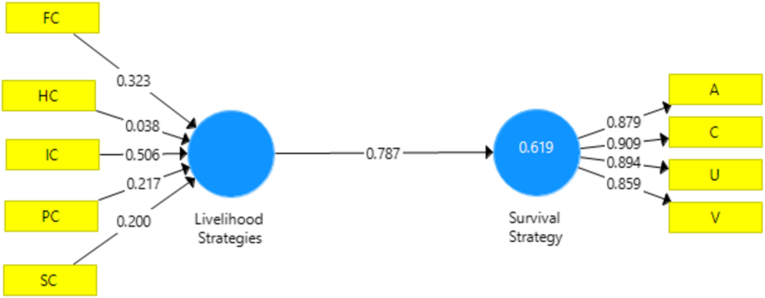
Outer picture/measurement model.

The outer Model’s testing in this study can be seen in Figure 2. It shows the PLS test results at the outer model stage in the variables. Tests are carried out on all of each variable. In this study, testing was carried out with a first-order pattern. The first latent variables are Livelihood Strategies and survival strategy-based VUCA. Livelihood Strategies have five indicators: human capital, social capital, financial capital, physical capital, and intellectual capital. The survival strategy variable consists of 4 sizes, namely strategy volatility, uncertainty, complexity, and ambiguity. The indicator criteria are valid and reliable in a constructive manner if they have a loading factor value greater than or equal to 0.5.

Figure 2 depicts the study’s outer model or measuring model, which includes formative measurement, construct variables, and livelihood strategies. VUCA serves as a proxy for a reflecting metric in survival strategies. A reflective measure with VUCA as a proxy is used in survival strategies.

Testing the outer Model in the first stage is the convergent validity value. Table 5 shows the result of convergent validity can be seen from the loading factor value. The loading factor value is valid; it must be more than 0.5. The complete convergent validity test results show that all variable dimensions have an outer loading value of more than 0.5, which means that the validity of each size is good. Discriminant validity can be measured using cross-loading values. The high cross-loading value (0.5) on the dimensions of certain variables compared to the dimension values of other variables means that the construct validity of these latent variables and dimensions is good.

**Table 5:** Convergent validity test.

	SL	Survival Strategy
A		0.879
C		0.909
V		0.859
U		0.894
FC	0.764	
HC	0.599	
IC	0.871	
PC	0.655	
SC	0.739	

Table 6 shows that cross-loading for each dimension of the variable is greater than the column for the dimensions of other variables. Each item has a value higher than the other construct. All have more variance with their measures than other constructs (Chin et al., 2016), and all indicators meet the discriminant validity test. It means that the variables meet the discriminant validity, and all dimension values are more significant than 0.5.

**Table 6:** Validity discriminant.

	SL	Survival Strategy
Survival strategy		
**VUCA**	0.787	0.886

The next measurement model is the Average Variance Extracted (AVE) value; the deal shows the amount of indicator variance contained by the latent variable. Measurement of validity and reliability, AVE, loading factor, communality, and composite reliability.

$$\text{VE}=\frac{\sum ⋌i^{2}\;var\;F}{\left(\sum ⋌i^{2}\right)var\;F+\sum \emptyset ii}$$

where: ⋌ is a loading factor; F is variance factor; ∅ii is variance error.

Table 7 shows AVE values greater than 0.5 also indicate good validity adequacy for latent variables. The calculation results show that all dimensions of the SL latent construct and the strategy VUCA latent construct have an AVE value of more than 0.5. With this result, all latent dimensions of each latent variable have good adequacy of construct validity. Table 7 shows that the SL and survival strategy dimensions as latent constructs have a Cronbach alpha value and composite reliability greater than 0.7. So that all variables are reliable.

**Table 7:** Construct validity and reliability.

	Cronbach’s Alpha	rho_A	Composite Reliability	Average Variance Extracted (AVE)
SL		1.000		
Survival Strategy	0.908	0.910	0.936	0.784

The last is testing the reliability of the construct in Table 7. The value of composite reliability measures the reliability of the construct; if the construct is reliable, if the value of the composite reliability is above 0.70, the indicator is said to be consistent in measuring its latent variables: SL dimensions, Cronbach’s alpha, and composite reliability in this research. Cronbach’s alpha and combined reliability value for all construct greater than 0.7, and it’s accepted (Hair et al., 1998). Therefore, we proceed with further analysis using all the calculated Cronbach’s alpha results constructs.

#### 4.1.2 Inner model or structural model testing phase

**Figure 3:**
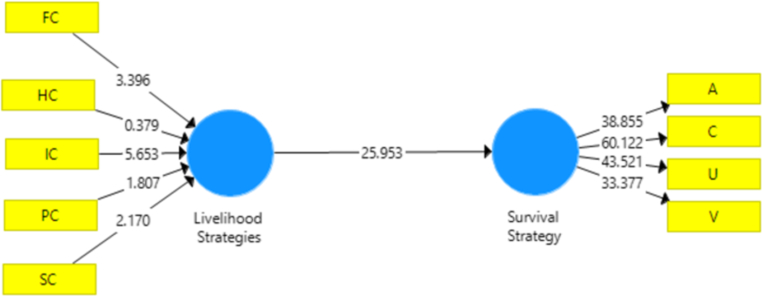
Inner testing/structural model.

The structural model stage aims to determine whether there is an influence between variables. Testing is done using the t-test. The variable is said to influence if the significance value of t is less than 0.05. The calculation results can be seen in the following Figure 3 below:

Hypothesis testing is done using the t-test. The variable is said to influence if the significance value of t is less than 0.05. The calculation results can be seen in the following table:

Table 8 shows the result of hypothesis testing for coefficient value of the Sustainable Livelihoods path to Survival Strategy is 0.787. The path coefficient is positive. This positive value means that the higher the Sustainable Livelihoods, the higher the Survival Strategy. And conversely, the lower the Sustainable Livelihoods, the lower the Survival Strategy. Then the coefficient value from the t-test results obtained a significance level of 0.000. The amount is smaller than 0.05, so there is a significant effect of the Sustainable Livelihoods on Survival Strategy. So that Ho is rejected and H1 is accepted.

**Table 8:** Hypothesis testing.

Relations between variables	Original Sample (O)	Sample Mean (M)	Standard Deviation (STDEV)	T Statistics (|O/STDEV|)	P Values
SL → Survival Strategy	0.787	0.798	0.030	25.953	**0.000**

#### 4.1.3 Coefficient of determination

The coefficient of determination describes the influence of exogenous variables on endogenous. Table 9 describes the results of the coefficient determination:

**Table 9:** Value of coefficient determination.

The relationship between variables	R Square	R Square Adjusted
Survival Strategy	0.619	0.617

The results showed that the magnitude of the influence of the Sustainable Livelihoods on Survival Strategy 0.619 was 61.9%. The remaining 0.431 or 43.1% was influenced by other variables not examined in this study.

### 4.2 Testing goodness of fit

#### 4.2.1 Predictive relevance

The last one is to find the Goodness of Fit (GoF) value. The goodness of fit indexes can be classified into different types, and one of them is the distinction between absolute and incremental fit indexes. Standardized root means square residual (SRMR) is most sensitive to factor covariance misspecification. Table 10 shows the Goodness of Fit measures. The SRMR (standardized basis mean square residual) observed correlation between the Model implied correlation matrix. It allows assessing the average magnitude of the discrepancies between observed and expected correlations as an absolute measure of the model fit criterion. The goodness of fit is less than 0.10 or 0.08, which is considered a good fit (Hu and Bentler, 2009). The SRMR calculation results show a value of 0.043. This value less than 0.10 (in a more conservative version) is considered a good fit.

**Table 10:** Value of coefficient determination.

	Saturated Model	Estimated Model
SRMR	0.043	0.043
d_ULS	0.083	0.083
d_G	0.097	0.097
Chi-Square	84.417	84.417
NFI	0.905	0.905

## 5 Discussion

### 5.1 Sustainability Livelihood ability to create a business strategy based on VUCA

The findings of the convergent validity test suggest that intellectual capital, with a loading value of 0.871, is the dimension that contributes the most to SL. On the other hand, human capital has the lowest value of 0.599. The most crucial thing women entrepreneurs must have concerning Sustainability Livelihood is intellectual capital. Meanwhile, complexity has a loading value of 0.909 based on survival strategy with VUCA as a proxy, while volatility has a loading value of 0.859. As a result, complexity stands out in the survival strategy with VUCA as a proxy that women entrepreneurs must be mindful of. Every entrepreneur wants their business to be long-term viable; business sustainability is defined as the ability of a company to continue operating for an extended period (Lebacq et al., 2012).

The research has conducted on 155 respondents who met the conditions, which included business ownership for a minimum of three years and monthly business revenue of 50 million rupiahs. 33.55% of respondents are over 30 years old, and the highest level of education is high school, with a proportion of 47%. The data showed that women entrepreneurs' human capital lacked business knowledge and experience that influenced business development.

During the COVID-19 pandemic, however, numerous hurdles threaten long-term viability. With VUCA as a proxy, the output reveals that human capital influences survival strategy; human resources should always be sharpened, especially in the corporate world. Even the most well-organized entity’s performance will be jeopardized if individuals with the required expertise, experience, and qualification are not recognized (Lamotte, 2012). Overall, a favorable relationship between human capital and livelihood methods is a valuable resource. Human capital is critical for developing high-quality new products to promote economic growth and long-term corporate competitiveness (Prasetyo and Kristanti, 2020). Human capital is measured by education, experience, skills, culture, and background and impacts the company’s personnel (Backman et al., 2016).

Furthermore, with a value of 0.739, social capital is an essential goal for entrepreneurs. It comprises participating in various business and entrepreneurial networks to receive new knowledge and relationships that can be used for their firm. Women entrepreneurs become more proactive in searching for information to uncover business prospects. To access business-related information, entrepreneurs might use both professional and informal media. Most micro-small-sized businesses, on the other hand, rely on more informal media such as Facebook, Instagram, and websites. The second factor is social capital, which influences the development of a survival strategy using VUCA as a proxy. Women entrepreneurs neither be able to exist alone as social beings nor will be able to stand alone as entrepreneurs. Friends, family, suppliers, internal-external stakeholders, and the community around their business are meaningful relationships for entrepreneurs to build and retain (Sallah and Caesar, 2020). The social capital of entrepreneurs encourages knowledge sharing about business expansion potential. Collaboration among entrepreneurs is advantageous because it increases marketing efforts.

Furthermore, some women entrepreneurs benefit from feedback from their networks, which helps them boost sales and acquire a competitive advantage over their competitors (Sallah and Caesar, 2020). According to Christy et al. (2022), social capital helps entrepreneurs succeed by allowing them to build trust among coworkers and business partners in exchange for support and growth. His research (Seo and Lee, 2019), indicated that a mix of discovery and local community support is critical for enhancing start-up firm performance in a fast-changing market and economic environment.

Financial capital was the third factor in the pyramid, and it had a significant impact on the VUCA strategy. Financial capital is required for the operation of a firm, and this capital can be obtained from financial institutions or personal savings. The value of financial capital in this study is 0.764. Financial capital is vital since it can help you create and develop your firm. The performance of new ventures is influenced by financial capital (Khan et al., 2019). Entrepreneurs must manage their budgets, save money, and obtain financial assistance in the form of microcredit or bank loans. Because many women entrepreneurs view financial resources as a significant obstacle, they are less likely to apply for debt financing due to the perceived difficulty. When it comes to acquiring financial resources, women entrepreneurs suffer far more discrimination and disadvantages than men (Kungwansupaphan and Leihaothabam, 2016). According to (R. R. Bennett and Dann, 2000), women entrepreneurs are less likely than men to obtain external finance, preferring to rely on internal resources such as personal savings or financial support from family and friends. Limited access and capital limits will impact a company’s long-term viability (Otoo et al., 2012).

Physical capital refers to the basic infrastructure and production inputs needed to support business, including production infrastructure and productive assets as vital capital that influence the company. Entrepreneurs continue to improve their products using technology such as constructing websites, and advertising via social media is a method employed by some entrepreneurs to establish their own business in physical capital with a value of 0.655, where asset ownership is crucial to sustaining the business. Physical capital transformation in the modern era is characterized by rapid technical advancements, whether in manufacturing machines or other supporting technologies such as marketing technology. Physical capital fulfillment is considered heavy by companies that need to keep up with the latest technologies to stay competitive. According to Taboada and Moya (2014), having greater physical capital affects the long-term viability of new ventures. Relationships between intellectual capital and other factors are also evident. Intellectual capital has a dominant value of 0.871 in our study, showing that respondents believe intellectual capital is crucial in corporate operations. Our informants, who are entrepreneurs, perform research on the legality of the enterprises they run, including intellectual property rights, BPOM, and other certifications required to operate their businesses. Several studies have looked into the relationship between IC, innovative activities, and competitiveness in businesses (Buenechea-Elberdin, 2017). The latter significant determinant of a firm’s competitive advantage is intellectual capital. Competitive advantage can be retained over time if intellectual capital is refreshed in response to changing external conditions, as suggested by dynamic capability theory (Crupi et al., 2020). Intellectual capital drives business development strategy, according to (Ibraimi and Rexhepi, 2011), while 6.5 percent of managers feel staff education has little impact on building strategy.

The results of hypothesis testing show that livelihood strategies impact women entrepreneurs' ability to devise survival strategies that will affect their ability to adapt, improve, and survive their businesses. The findings revealed that five aspects of women’s livelihood strategies impact survival strategy business, using VUCA as a proxy. Welbourne and Pardo-del-Val (2009) define organizational capital as “the entirety of a company’s abilities that enable it to meet market criteria,” implying that entrepreneurs are responsible for building corporate capital. Firm profitability is favorably associated with business performance when organizational capital increases as business capital in this study (Aminu et al., 2015). During Covid-19, women’s empowerment was modeled.

The outcomes of the FGD are explained in this section. With VUCA as a proxy, apply FGD to construct a model in livelihood strategies to survival strategies for women entrepreneurs. Table 11 summarizes the findings of the focus group, which revealed a comprehensive model of sustainability for women entrepreneurs.

**Table 11:** Focus group discussion result.

The vulnerability caused by COVID-19	
Aspects	Output
Shock Event	▪ Sudden events that impact livelihood security, particularly in business. ▪ Businesses may close because of accommodations, food, and educational services affected by changed customer behavior, especially the physical distancing and mandated operational restrictions. ▪ Businesses may close because they were already at risk financially before the crisis. ▪ They experienced a massive decline in revenue. ▪ Limited access to new skills & knowledge ▪ Lack of financial and business services ▪ Lack of information ability ▪ Lack of innovation and creativity ability ▪ Lack sufficient working capital to run their entrepreneurship activities, so in this regard, their financial assets portfolio is low. ▪ Stressful/Mental health ▪ Fatigue, at-home responsibilities ▪ Deficit saving ▪ Loss of household assets ▪ Liquidate assets.
**Important capital to survive**	
**Pentagon Assets Unit**	**Output**
Human Capital	Having a skill and a business strategy is very important for every business to maintain business continuity.
Social Capital	Having relationships and the support of the closest people is very important in maintaining business continuity.
Financial Capital	Capital loans from the government, in this case, greatly assisted the capital of women entrepreneurs. During the pandemic era, payment relief in deferred payments was also beneficial for women entrepreneurs to survive. An affirmative movement is needed to make it easier for women to obtain loans from financial institutions, banks, and non-banks. They are changing the payment system with clients to maintain the company’s cash flow.
Physical Capital	Keep abreast of technological developments, especially those related to their business needs. Understand what and how much investment in production infrastructure to keep their business afloat.
Intellectual Capital	Having a good reputation and having non-physical assets such as Intellectual Property, Certification helps the smooth running of a business and business development.
**Support System**	
**Support** **System**	**Output**
Structures and processes	Institutions, organizations, policies, and legislation that shape livelihoods, particularly in women entrepreneur business Policies, legal instruments, and enforcement can remove constraints to developing women’s entrepreneurship during the pandemic. Government agencies, NGOs, and the private sector can provide technical support to women entrepreneurs.

### 5.2 Modeling women’s empowerment during Covid-19

In this section, we explain the results of FGD. We use FGD to create a livelihood strategy to survival strategy for women entrepreneurs with VUCA as a proxy. The obtained results of FGD:1) Vulnerability caused by pandemic covid 19; 2) Structures and processes that are needed as a strong support system; 3) Five capitals that build livelihood sustainability; 4) Survival Strategy. Those 4 components as part of the integrative model. Results of FGD which revealed the comprehensive Model of sustainability for women entrepreneurs, were tabulated in Table 11.

This research will provide helpful information and advice on entrepreneurship expertise and the most critical aspects affecting women entrepreneurs' ability to survive their businesses, specifically how they might raise their welfare using livelihood strategies during the pandemic. COVID-19. This research focuses on the environment and state of vulnerability created by the COVID-19 epidemic. Women entrepreneurs face a wide range of issues. The challenges above must undoubtedly be handled with five types of capital controlled by entrepreneurs based on lifestyle methods. The team proposed a locally relevant survival strategy women entrepreneurship model-based VUCA as a proxy of survival strategy based on quantitative analysis and focus group discussions.

### 5.3 Vulnerability

The first approach for VUCA is vulnerability; entrepreneurs should look into business adjustments in response to pandemics and focus on adequate resources. According to the Schumpeterian Model, economic downturns destroy less innovative enterprises while allowing more innovative firms to survive and develop (Jung and Kim, 2018).

### 5.4 Uncertainty

Entrepreneurs must comprehend the strategy by focusing on a broader understanding of environmental change and gathering relevant information for the maturation of future systems to overcome uncertainty.

### 5.5 Complexity

Clarifying the strategy can help to reduce the complexity. Entrepreneurs must, in particular, develop a more extensive collaboration by redesigning the business to be more productive and efficient. It is accomplished through collaboration with like-minded entrepreneurs.

### 5.6 Ambiguity

**Figure 4:**
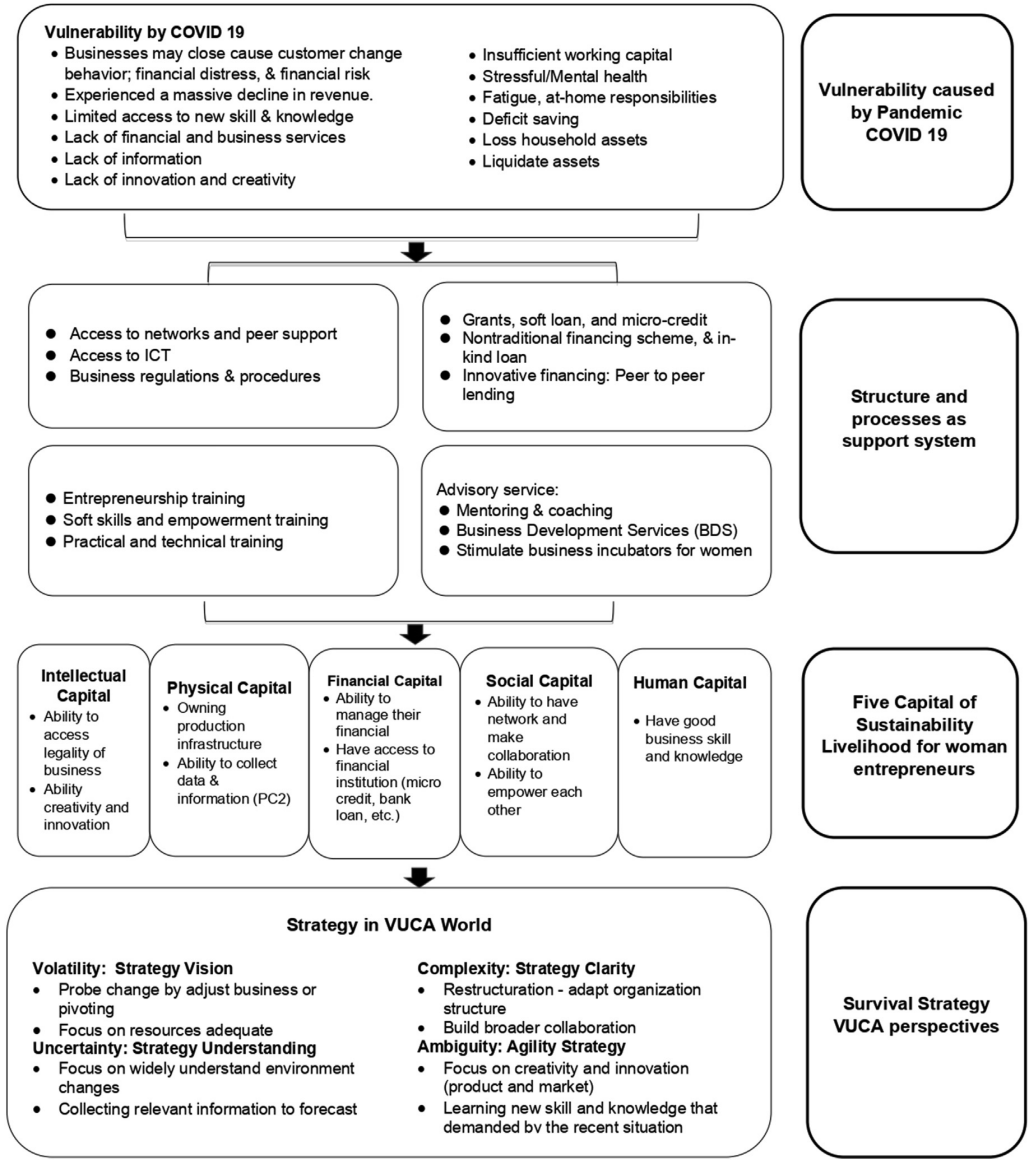
Women entrepreneurs livelihood strategy & strategy survival business based on VUCA

With a focus on building creativity and innovations in both products and markets, entrepreneurs can overcome uncertainty in this research. It must also learn how to do digital marketing, place ads on social media, write content, and learn new skills required by pandemic situations (Radomska et al., 2019). found that networking is one of the most critical factors influencing ambidextrous activities. Figure 4 depicts strategies that can be developed based on VUCA. Over the last several decades, there has been a lot of change, debate, and advancement in the theory and practice of many approaches to sustainability, especially when it comes to women entrepreneurs and the current situation with Pandemic COVID-19. This paradigm should help women entrepreneurs adjust to the volatility, unpredictability, complexity, and ambiguity of today’s world, both theoretically and practically. Adaptive solutions based on involvement, empowerment, current knowledge, technology, financial services, and improvements in government policies are used to achieve sustainable livelihood goals (DFID, 1999). Local people’s existing capital in five capital-based assets: natural, financial, physical, human, and social capital is often the emphasis of sustainable livelihood techniques (DFID, 1999). The framework structure integrates and tailors a well-used and approved sustainable livelihood framework to illustrate how women entrepreneurs adopt tactics to adapt and sustain their businesses.

The respondents came from throughout Indonesia, and they are of varied ages and educational levels. Most respondents (33%) are between 30 and 35, and high school graduates account for nearly half of all respondents (47%). According to the findings, having business skills is important for the continuity of the business being run, always finding out what is required to develop a business, building relationships such as following the business community, and always willing to learn about developing trends to increase product competitiveness, implementing regulations such as Intellectual Property Rights, and required certification.

Figure 4 shows a model of women entrepreneurs' livelihood Strategy & Strategy Survival Business Based on VUCA. Information collected by FGD which explains COVID-19 as a complex worldwide social phenomenon has shown how vulnerable small firms are, especially for women entrepreneurs, and the importance of resilience for SMEs. During a pandemic, women entrepreneurs face several issues, including Business closures, customer behavior changes, financial distress, and financial risk, limited access to new skills and knowledge, a lack of financial and business services, a lack of information, a lack of innovation and creativity ability, a lack of sufficient working capital, stressful/mental health, fatigue, at-home responsibilities, deficit saving, and the loss of household assets are just a few of the challenges that women entrepreneurs face. Human Capital, Social Capital, Physical Capital, Financial Capital, and Intellectual Capital were created to analyze the business livelihood of women entrepreneurs based on data acquired by questionnaire. Capital must be available and capable of devising a plan to deal with recent developments. In addition, the government, the private sector, and non-governmental organizations (NGOs) must collaborate to accelerate the transformation of processes and activities to adapt and respond rapidly enough to continue in business. Consular services and business assistance are accessible through the Ministry of Small and Medium Business Cooperatives and soft skills, practical, and technical training.

## 6 Conclusions

The goal of this research is to create an integrative approach for helping women entrepreneurs to continue in business in the face of the COVID-19 epidemic. The following are the research’s findings: The research’s findings reveal that the pentagon asset from the livelihood strategy is an excellent supportive factor for women entrepreneurs seeking to maintain their businesses during Covid-19. In other words, they are demonstrating that women entrepreneurs can improve their quality of life by developing a livelihood strategy during COVID-19. Second, the five capitals that women entrepreneurs have accessed have indirectly become essential supporters in developing new VUCA-based business strategies. A solution for women entrepreneurs to survive and grow their firms is a sustainability business-based integrative sustainable livelihood model. The ability to access five elements of sustainable livelihood Pentagon Assets in Sustainable livelihoods: 1) Human Capital; 2) Social Capital; 3) Financial Capital; 4) Physical Capital; 5) Intellectual Capital has a positive relationship with the ability to create a business strategy based on VUCA perspective, according to PLS output. An integrative model based on the Sustainable Livelihood and VUCA perspectives can be used as a strategy in women entrepreneur empowerment to address the various uncertainties every woman entrepreneur in Indonesia recently during COVID-19. As a result of the preceding debate, women entrepreneurs must manage their businesses more creatively to remain competitive. Due to the COVID-19 pandemic, lifestyle changes and consumer trends must be addressed. It can be accomplished through acquiring the most up-to-date knowledge, polishing skills and identifying possibilities, developing new ideas, and forming partnerships or expanding one’s network.

## 7 Future research and implication

This study implies the academic community as well as the government. First, the findings illuminate the impact of the COVID-19 epidemic on women entrepreneurs' ability to survive. Second, these findings have significant implications for education in establishing a sustainable livelihoods development model based on women entrepreneurs' VUCA approach. Third, this is significant for the government since it requires participation and support in the form of more convenient and accessible facilities for entrepreneurs; additionally, it is expected that the government can simplify regulation for the advantage of women-owned SMEs. It’s crucial to keep in mind the study’s limitations. First, this study only looked at women entrepreneurs; future studies could look into men or both. Second, the sample only includes one country; the next researcher can expand the selection to include more countries. More research into the Model used in this study could lead to new models and strategies for surviving in an unpredictable world.

## Declarations

### Author contribution statement

Achsania Hendratmi and Puji Sucia Sukmaningrum: Conceived and designed the experiments; Performed the experiments; Analyzed and interpreted the data; Contributed reagents, materials, analysis tools or data; Wrote the paper.

Tri Siwi Agustina and Mega Ayu Widayanti: Performed the experiments; Contributed reagents, materials, analysis tools or data.

### Funding statement

Achsania Hendratmi was supported by kementerian riset, teknologi dan pendidikan tinggi [645/UN3.14/PT/2020].

### Data availability statement

Data will be made available on request.

### Declaration of interests statement

The authors declare no conflict of interest.

### Additional information

No additional information is available for this paper.
